# That's my story, and I'm sticking to it—an update on *B. burgdorferi* adhesins

**DOI:** 10.3389/fcimb.2014.00041

**Published:** 2014-04-03

**Authors:** Catherine A. Brissette, Robert A. Gaultney

**Affiliations:** Department of Basic Sciences, University of North Dakota School of Medicine and Health SciencesGrand Forks, ND, USA

**Keywords:** *Borrelia burgdorferi*, Lyme disease, bacterial adhesins, extracellular matrix, fibronectin, decorin, integrins

## Abstract

Adhesion is the initial event in the establishment of any infection. Borrelia burgdorferi, the etiological agent of Lyme disease, possesses myriad proteins termed adhesins that facilitate contact with its vertebrate hosts. *B. burgdorferi* adheres to host tissues through interactions with host cells and extracellular matrix, as well as other molecules present in serum and extracellular fluids. These interactions, both general and specific, are critical in the establishment of infection. Modulation of borrelial adhesion to host tissues affects the microorganisms's ability to colonize, disseminate, and persist. In this review, we update the current knowledge on structure, function, and role in pathogenesis of these “sticky” *B. burgdorferi* infection-associated proteins.

## Introduction

Spirochetes are unique among bacteria in their extraordinary ability to invade, disseminate, and persist in their hosts. There is no invasion, dissemination, or persistence, however, without the ability to first adhere to host cells and surfaces. Adhesion is the initial event in the establishment of any infection. Failure to bind with sufficient strength to host tissues results in the bacterial pathogen's clearance by host innate immune mechanisms. Binding too strongly to host tissues can be detrimental to the pathogen, however, by hindering the ability of the bacterium to disseminate throughout the host. To facilitate this dynamic attachment, bacteria possess proteins called adhesins that enable binding to specific host molecules. A major adhesive target of bacterial pathogens is the extracellular matrix (ECM), a complex network of proteins and carbohydrates between and beneath cells. This supportive lattice typically functions in eukaryotic cell movement, development, and growth (Lodish and Darnell, 2000).

Lyme disease is the most common arthropod-borne disease in the northern hemisphere, and is caused by species of the *Borrelia burgdorferi* sensu lato complex, which includes *B. burgdorferi* sensu stricto (henceforth *B. burgdorferi*), *B. garinii*, *B. afzelii*, *B. spielmanii*, and *B. bavariensis* (Margos et al., 2011; Stanek and Reiter, 2011; Stanek et al., 2012). The Lyme disease spirochete can infect immunocompetent humans and other vertebrates for extensive periods of time, even for the animal's lifetime (Moody et al., 1990; de Souza et al., 1993; Steere, 2001; Miller et al., 2003; Stanek et al., 2012). As an extracellular pathogen, *B. burgdorferi* interacts with cells, tissues, and components of the ECM. *In vivo*, *B. burgdorferi* is frequently found associated with connective tissues (Kornblatt et al., 1984; De Koning et al., 1987; Barthold et al., 1991, 1992a,b, 1993; Defosse et al., 1992; Häupl et al., 1993; Pachner et al., 1995; Franz et al., 2001; Coburn et al., 2002; Cadavid et al., 2003; Cabello et al., 2007) and detected in infected cartilaginous tissues, such as skin and joints (Kornblatt et al., 1984; Schwan et al., 1988; Sinsky and Piesman, 1989; Barthold et al., 1991, 1992b; Melchers et al., 1991; Berger et al., 1992; Defosse et al., 1992; Schwartz et al., 1992; Shih et al., 1992; Pachner et al., 1995; Coburn et al., 2002; Liveris et al., 2002; Miller et al., 2006; Bykowski et al., 2007). Indeed, the ability of *B. burgdorferi* to invade collagenous tissue has been suggested as a possible mechanism of immune evasion (Cadavid et al., 2003; Barthold et al., 2006; Cabello et al., 2007). *B. burgdorferi* interactions with the host ECM are therefore likely critical in both the spirochete's pathogenesis as well as its persistence in mammals.

*B. burgdorferi* binds various components of the ECM, including glycosaminoglycans (GAGS), fibronectin, decorin, collagen, laminin, and integrins. In addition, *B. burgdorferi* adheres to numerous host cell types and binds components of host serum and extracellular fluids, such as plasminogen and complement regulators (Table 1). A number of *B. burgdorferi* adhesins were recently reviewed by Antonara et al. (2011). However, considerable research into the functions and biomolecular interactions of these adhesins has taken place since then, including the identification of novel Lyme spirochete adhesins. In this review, we provide an update on *B. burgdorferi* adhesins, focusing specifically on the bacterial factors that interact with components of the ECM, plasminogen, and complement regulators. For readers interested in adhesins involved in spirochete/tick interactions, please refer to the recent review by Kung et al. (2013). We would also refer the reader to the excellent discussion of novel *in vivo* imaging techniques and their use in delineating the roles of *B. burgdorferi* adhesins in a infectious mouse model in the work of Coburn et al. (2013).

**Table 1:** ***B. burgdorferi* adhesins**.

**Function**	**Adhesin (genetic locus)**	**Comments**	**References**
**FIBRONECTIN BINDING**			
	BBK32 (*bbk32*)	Also binds GAGs; mutants attenuated in mice; role in vascular interactions in mice	Probert and Johnson, 1998; Probert et al., 2001; Kim et al., 2004; Raibaud et al., 2005; Fischer et al., 2006; Li et al., 2006; Seshu et al., 2006; Norman et al., 2008; Hyde et al., 2011; Chan et al., 2012; Lin et al., 2012; Moriarty et al., 2012
	RevA (*bbM27, bbP27*)	*bbM27* attenuated in mice; early antigen of Lyme disease; not involved in vascular interactions in mice	Gilmore and Mbow, 1998; Carroll et al., 2001; Mbow et al., 2002; Brissette et al., 2009a, 2010; Lin et al., 2012; Moriarty et al., 2012; Floden et al., 2013
	RevB (*bbC10*)	Attenuated in mice; not involved in vascular interactions in mice	Brissette et al., 2009a, 2010; Lin et al., 2012; Moriarty et al., 2012
	BB0347 (*bb0347*)	Not involved in vascular interactions in mice	Moriarty et al., 2012; Gaultney et al., 2013
	CspA (*bbA68*)	Also binds complement regulator proteins, plasminogen, laminin, others	Hallstrom et al., 2010
	CspZ (*bbH06*)	Also binds complement regulator proteins, laminin	Hallstrom et al., 2010
**GAG BINDING**			
	DbpA/B (*bbA24, bbA25*)	Bind decorin and dermatan sulfate; attenuation of infection in mice	Fischer et al., 2003; Shi et al., 2006; Blevins et al., 2008; Shi et al., 2008a,b; Benoit et al., 2011; Hyde et al., 2011; Chan et al., 2012; Lin et al., 2012; Wang, 2012; Imai et al., 2013; Morgan and Wang, 2013
	Bgp (*bb0588*)	Attenuated in mice	Parveen et al., 2006; Lin et al., 2012
	BbhtrA (*bb104*)	Binds and cleaves aggregan; first cell-associated protease described in *B. burgdorferi*	Coleman et al., 2013; Kariu et al., 2013; Russell and Johnson, 2013; Russell et al., 2013
**LAMININ BINDING**			
	BmpA (*bb0382*)	Potential role in arthritis	Pal et al., 2008; Yang et al., 2008; Verma et al., 2009
	ErpX (on lp56 of B31)		Brissette et al., 2009c
	CspA (*bbA68*)	Also binds complement regulator proteins, plasminogen, fibronectin, others	Hallstrom et al., 2010
	CspZ (*bbH06*)	Also binds complement regulator proteins, plasminogen, fibronectin, others	Hallstrom et al., 2010
**COLLAGEN BINDING**			
			Zambrano et al., 2004
	CspA (*bbA68*)	Also binds complement regulator proteins, plasminogen, fibronectin, others	Hallstrom et al., 2010
	CspZ (*bbH06*)	Also binds complement regulator proteins, laminin, fibronectin	Hallstrom et al., 2010
**INTEGRIN BINDING**			
	P66 (*bb0603*)	Binds α1β3; also porin; essential for infection of mice	Antonara et al., 2007; Behera et al., 2008; Lafrance et al., 2011; Ristow et al., 2012
	BB0172 (*bb0172*)	Binds α3β1; MIDAS motif	Wood et al., 2013
**PLASMINOGEN BINDING**			
	BB0337(*bb0337*)	Enolase; moonlighting protein	Floden et al., 2011; Nogueira et al., 2012; Toledo et al., 2012
	OspC (*bbB19*)	Required for establishment of infection	Grimm et al., 2004; Tilly et al., 2006; Antonara et al., 2007, 2010; Tilly et al., 2007; Xu et al., 2008; Lin et al., 2012; Onder et al., 2012
	CspA (*bbA68*)	Also binds complement regulator proteins, laminin, others	Hallstrom et al., 2010; Hammerschmidt et al., 2014
	BBA70 (*bbA70*)		Koenigs et al., 2013
	ErpA/C/P (*bbP38, bbI39, bbN38*)	ErpA mutants have slight attenuation in mice	Alitalo et al., 2002; Brissette et al., 2009b; Lin et al., 2012
**COMPLEMENT REGULATOR BINDING**			
			Kraiczy and Stevenson, 2013
	CspA (*bbA68*)	Binds FH, FHL-1	Hellwage et al., 2001; Alitalo et al., 2002; Metts et al., 2003; Hallstrom et al., 2010; Caesar et al., 2013b; Hammerschmidt et al., 2014
	CspZ (*bbH06*)	Binds FH, FHL-1	Hartmann et al., 2006; Rogers and Marconi, 2007; Kraiczy et al., 2008; Rogers et al., 2009
	ErpA/C/P (*bbP38, bbI39, bbN38*)	Bind FH, different binding affinities for FHR1, 2, 5	Kenedy et al., 2009; Hammerschmidt et al., 2012; Bhattacharjee et al., 2013; Caesar et al., 2013a; Meri et al., 2013

*Abbreviations: Bgp, Borrelia glycosaminoglycan-binding protein; Bmp, Borrelia membrane protein; Dbp, decorin binding protein; Erp, OspEF-related protein; FHL, factor H-like; FHR-1, factor H related-1; GAG, glycosaminoglycan*.

### Fibronectin binding proteins

Fibronectin is a large plasma glycoprotein and a prominent component of the ECM and host serum (Romberger, 1997). In the uninfected host, fibronectin has many important roles including involvement in cell adhesion, cell migration, cell-cell signaling, ECM remodeling, and cell proliferation, survival and differentiation (Hynes, 1999; Pankov and Yamada, 2002; Midwood et al., 2006). Many bacterial pathogens interact with fibronectin, including several pathogenic spirochetes (Fitzgerald et al., 1984; Umemoto et al., 1993; Kopp et al., 1995; Grab et al., 1998; Probert and Johnson, 1998; Merien et al., 2000; Probert et al., 2001; Cameron et al., 2004; Schwarz-Linek et al., 2004, 2006; Choy et al., 2007; Lee and Choi, 2007; Stevenson et al., 2007; Atzingen et al., 2008; Brinkman et al., 2008; Hauk et al., 2008; Brissette et al., 2009a; Bamford et al., 2010; Henderson et al., 2011). *B. burgdorferi* binds to fibronectin *in vitro* (Kopp et al., 1995; Grab et al., 1998; Probert and Johnson, 1998). Additionally, soluble fibronectin or anti-fibronectin antibodies inhibit interactions between *B. burgdorferi* and cultured endothelial cells and their secreted matrices (Szczepanski et al., 1990; Kopp et al., 1995). *In vivo*, fibronectin interactions are critical in the initiation of borrelial interactions with the microvasculature (Norman et al., 2008). To date, there have been five fibronectin binding proteins identified in *B. burgdorferi*: BBK32, RevA/B, BB0347, and CspA (CRASP-1).

#### BBK32

BBK32, a 47 kDa protein encoded on linear plasmid 36 (lp36), binds the glycosaminoglycans heparin sulfate and dermatan sulfate, in addition to fibronectin. BBK32 was the first fibronectin-binding protein identified in *B. burgdorferi* (Probert and Johnson, 1998; Probert et al., 2001). BBK32 deletion mutants demonstrate a slight, but significant, defect in infectivity in the mouse (Li et al., 2006; Seshu et al., 2006). BBK32 has sequence similarities with fibronectin-binding adhesins of Gram-positive pathogens such as *Staphylococcus aureus* and the streptococci. Indeed, BBK32 interacts with fibronectin via the “tandem beta zipper” mechanism that is common for fibronectin-binding adhesins of streptococci (Kim et al., 2004; Raibaud et al., 2005). Several recent studies have investigated the functions of BBK32 in *B. burgdorferi* pathogenesis (Hyde et al., 2011; Wu et al., 2011; Chan et al., 2012; Moriarty et al., 2012; Ranka et al., 2013).

Recently, a whole body imaging system was utilized to examine the role of BBK32 in dissemination throughout the murine host (Hyde et al., 2011). Luciferase-expressing *B. burgdorferi* lacking BBK32 were injected into mice. The authors demonstrated a decrease in the infectious load of *bbk32* mutants at days 4 and 7 compared to the parental strain. Dissemination defects were more pronounced at lower inocula, and disappeared with higher doses of bacteria and with later time points (14 days post-inoculation). These data are consistent with the infectivity phenotypes observed in previous studies, which implied that BBK32 is important in the earlier stages of infection (Li et al., 2006; Seshu et al., 2006). The imaging results correlated well with quantitative PCR data, suggesting that whole body imaging may be a useful tool to discern subtle differences in colonization and dissemination with other borrelial mutants. These studies are also significant as the first use of bioluminescent imaging in mice with *B. burgdorferi* mutants (Hyde et al., 2011).

Elegant studies recently have demonstrated the involvement of BBK32 in adhesion to the vasculature through the use of intravital microscopy (Moriarty et al., 2012). BBK32-fibronectin interactions were shown to be important for the initial tethering of the bacterium to the endothelium, a process termed “molecular braking.” This transient slow- down allowed for more stable interactions between BBK32 and glycosaminoglycans, leading to dragging of the bacterium along the endothelium, stationary adhesion of the bacterium to the endothelium, transmigration, and, finally, escape from the vasculature in a manner akin to the diapedesis of leukocytes (Muller, 2009). BBK32-deficient bacteria had significantly reduced interactions per minute, but still adhered to the endothelium, signifying that other adhesins are involved in the initial tethering step. The authors looked at the potential role of other fibronectin-binding proteins; gain of function studies were performed with a high passage nonadherent strain of *B. burgdorferi* overexpressing RevA, RevB, or BB0347. None of these proteins acted as vascular adhesins in fluid shear forces under the conditions of this experiment (Moriarty et al., 2012), suggesting additional borrelial adhesins (fibronectin binding or otherwise) are involved.

Another study evaluated a *B. burgdorferi* strain, N40D10/E9, which naturally lacks *bbk32* and has decreased binding to Vero (epithelial) cells. This strain disseminated at lower doses than the *B. burgdorferi* type strain, which does possess BBK32. The authors postulate this difference may be due to the lack of binding of the bacteria to epithelial cells at the site of inoculation (Chan et al., 2012). An important point gleaned from the above two studies is that while adhesion is a critical virulence factor for *B. burgdorferi*, that adhesion must be modulated to allow the bacterium to disseminate, either hematogenously or through the tissues. Adhering too strongly to tissues or cells is likely just as detrimental as not adhering at all.

*B. burgdorferi* is generally considered an extracellular pathogen, although there are numerous studies demonstrating at least transient intracellularity (Ma et al., 1991; Klempner et al., 1993; Dorward et al., 1997; Livengood and Gilmore, 2006). Recently, specific integrins were shown to be involved in *B. burgdorferi* invasion of mouse fibroblasts and human umbilical vein endothelial cells (HUVEC) (Wu et al., 2011). Since these integrins interact with fibronectin, Wu et al. examined the role of BBK32 as a bridge in the uptake of *B. burgdorferi*. BBK32 was not involved in the invasion of *B. burgdorferi* into murine fibroblasts, human fibroblasts or HUVECs, suggesting other components (such as additional fibronectin binding proteins) of *B. burgdorferi* interact with integrins to trigger uptake (Wu et al., 2011).

However, BBK32 protein fragments were used for the successful intracellular targeting of nanoparticles into epithelial cells, based on a Hepatitis B core protein system. Hepatitis B virus core is a 21 kDa protein which self-assembles into 35 nm particles (Wynne et al., 1999). Using region 130–166 of BBK32 (fibronectin binding region) fused into the major immunodominant region of the Hepatitis B core antigen, the authors demonstrated both fibronectin binding and uptake of the engineered nanoparticles into baby hamster kidney cells (Ranka et al., 2013). Targeting nanoparticles to cells or tissues of interest using a bacterial adhesin might be an efficient approach for drug delivery.

#### RevA/B

RevA is a 19 kDa surface protein encoded on the circular plasmid 32 (cp32) family of plasmids. RevB is encoded on cp9, and shares 28% overall amino acid sequence identity with RevA. RevA expression is upregulated in the mammal compared to the tick vector, and its expression pattern and surface exposure suggest a potential role in *B. burgdorferi* pathogenesis (Gilmore and Mbow, 1998; Carroll et al., 2001; Mbow et al., 2002; Brissette et al., 2009a). Both RevA and RevB were shown to bind fibronectin *in vitro*, and anti-RevA antibodies block binding of whole *B. burgdorferi* to fibronectin (Brissette et al., 2009a).

Recently, the potential for RevA to serve as a diagnostic tool or vaccine was evaluated. RevA is expressed early upon mammalian infection, and patients in various stages of Lyme disease, including patients with erythema migrans, exhibited antibodies to this protein (Brissette et al., 2010). In contrast, RevB lacked utility as a potential diagnostic marker; patients rarely possessed antibodies to RevB, probably because cp9 is a plasmid often lost even in infectious wild-type *Borrelia* (Brissette et al., 2010). In mice, there was a rapid IgM response to RevA, which remained steady over months, and was followed by a variable IgG response. Polyclonal rabbit anti-RevA antibodies were bactericidal *in vitro*, but serum from vaccinated mice was not. Mice vaccinated with RevA were not protected when exposed to *B. burgdorferi* by needle or tick bite (Floden et al., 2013); however, passive immunization with the bactericidal rabbit anti-RevA antibodies did prevent infection. The reason for this disparity is not known; a bolus of anti-RevA antibody concentrated near the site of infection may be necessary for the observed bactericidal effect.

In addition to their bactericidal effects, antibodies against adhesins can block critical interactions between bacteria and their host. Schmit and colleagues investigated the ability of various monoclonal antibodies against *B. burgdorferi* outer surface proteins to block binding of the spirochete to human cells. Monoclonal antibodies against RevA did not prevent binding of *B. burgdorferi* to HUVECS or H4 neuroglial cells (Schmit et al., 2011). Since monoclonal antibodies were used, it is possible that polyclonal antibodies recognizing multiple epitopes of RevA could block binding of the bacterium to cells. Indeed, a polyclonal antibody against RevA prevents binding of *B. burgdorferi* to immobilized fibronectin (Brissette et al., 2009a). However, nonadherent *B. burgdorferi* overexpressing RevA did not exhibit increased binding to the vascular endothelium *in vivo*, which suggests this adhesin is not involved in endothelial cell interactions (Moriarty et al., 2012). RevA's role in pathogenesis remains to be elucidated; however, a *revA* mutant uncovered in a global signature-tagged mutagenesis study did demonstrate an infectivity defect (Lin et al., 2012). It is likely that many borrelial adhesins have distinct tissue and cell type tropisms, as well as overlapping and redundant functions.

#### BB0347

The chromosomally-encoded protein BB0347 was recently confirmed to bind fibronectin by two independent studies (Moriarty et al., 2012; Gaultney et al., 2013). Previously, BB0347 had been annotated as a putative fibronectin binding protein due to sequence similarity with other such proteins (Fraser et al., 1997). Gaultney et al. demonstrated that BB0347 was expressed and surface exposed, and that mice infected with live *B. burgdorferi* produced anti-BB0347 antibodies (Gaultney et al., 2013). The importance of BB0347 in murine infection has not been thoroughly tested; however, overexpression of this protein does not restore the ability of noninfectious, nonadherent *B. burgdorferi* to bind host vasculature (Moriarty et al., 2012). Additionally, results suggest that BB0347 has a higher dissociation constant (K_D_) than BBK32, suggesting this protein does not bind fibronectin as strongly (Moriarty et al., 2012; Gaultney et al., 2013). Ultimately, the role of BB0347 in *B. burgdorferi* pathogenesis is still largely unknown, and further research is needed to determine the functionality of this protein in the Lyme spirochete.

#### CspA (CRASP-1)

CspA, also known as Complement Regulator Acquiring Surface Protein-1 (CRASP-1), is a factor H binding protein important in *Borrelia* resistance to complement (Kraiczy et al., 2001b; Brooks et al., 2005). Like many *B. burgdorferi* adhesins, CspA has multiple functions and binding partners (Kraiczy et al., 2001a; Hallstrom et al., 2010). Recent work by Hallstrom et al. demonstrated that CspA binds to fibronectin *in vitro*, as well as to human bone morphogenic protein 2 (BMP2), plasminogen, collagens I, III, and IV, and laminin. CspZ (CRASP-2), another factor H binding protein, also bound fibronectin and laminin *in vitro* (Hallstrom et al., 2010).

### Proteoglycan and GAG binding proteins

Proteoglycans are a diverse group of highly viscous macromolecules with a core protein and attached polysaccharide chains called glycosaminoglycans, or GAGs (Lodish and Darnell, 2000). Glycosaminoglycans, including chondroitin sulfate, dermatan sulfate, keratin sulfate, heparin, heparin sulfate, and hyaluronan, are long unbranched polysaccharides comprised of a repeating disaccharide unit (Varki, 2009). *B. burgdorferi* binds to both proteoglycans and their GAG chains through a number of proteins, including decorin-binding proteins A and B (DbpA/B), *Borrelia* glycosaminoglycan- binding protein (Bgp), and BBK32 (Parveen and Leong, 2000; Fischer et al., 2003, 2006).

#### Decorin-binding protein A/B

Decorin is a small leucine rich proteoglycan that associates with collagen; it possesses a collagen binding core protein and single GAG chain, either dermatan sulfate or chondroitin-6 sulfate (Danielson et al., 1997; Keene et al., 2000; Zhang et al., 2006). Decorin is widely expressed, and has important functions not only as a structural molecule of the ECM, but also as a cell signaling molecule influencing growth, differentiation, and inflammation (Dugan et al., 2006). Decorin also interacts with fibrinogen, allowing interaction with the hemostasis and thrombosis cascades (Dugan et al., 2006; Seidler, 2012). *B. burgdorferi* has two decorin-binding proteins that recognize decorin and other proteoglycans with GAG chains (Guo et al., 1995, 1998; Leong et al., 1998; Parveen et al., 1999). The genes encoding decorin-binding proteins A and B (*dbpA/B*) are encoded in a bicistronic operon on lp54. Mutants deficient in decorin- binding proteins are still infectious; however, *dbpA/B* deficient bacteria are impaired in both colonization of various tissues and in persistent infection (Shi et al., 2006, 2008a,b; Weening et al., 2008). Decorin deficient mice are resistant to *B. burgdorferi*, exhibiting fewer bacteria in joints upon infection as well as less severe arthritis than that induced in wild type mice (Brown et al., 2001). These data highlight the importance of decorin interactions to the establishment of disseminated infections by *B. burgdorferi*.

The recently solved structure of DbpA lends insight into the biophysical interaction of this protein with GAGs (Wang, 2012). DbpA was shown to be a helical bundle protein with 5 helices and a strong hydrophobic core. The authors demonstrated that a patch composed of basic amino acids is formed by portions of two helices and two flexible linkers. Residues K163, K170, and R166 formed a basic stretch on helix 5, reminiscent of motifs present in other GAG-binding helices. Heparin titration of DbpA showed that perturbed residues were in the linker domain between helices 1 and 2, demonstrating an important role for this linker in binding GAGs. Dermatan sulfate binds with a lower affinity than heparin, but still binds DbpA (Wang, 2012). In a separate study, the heparin binding epitope was identified as a BXBB motif (*B = basic* amino acid) in the linker between helices 1 and 2 (Morgan and Wang, 2013).

Based on the structural data, it seems likely that DbpA allelic variation, particularly in the GAG binding region, would affect the ability of *B. burgdorferi* to bind to decorin, GAGs, and mammalian cells. In gain of function studies, B314, a noninfectious and nonadherent *B. burgdorferi* strain, was used to examine the binding characteristics of various DpbA alleles. Expression of various alleles of DbpA in the nonadherent background allowed adhesion to epithelial but not endothelial or glial cells (Benoit et al., 2011). The authors also demonstrated that decorin and dermatan sulfate binding were separable, suggesting different binding sites on the DbpA protein for decorin and dermatan sulfate. The DbpA protein from strain N40 showed reduced adhesion to GAGs *in vitro*; however, the N40 strain caused disseminated infection *in vivo* (Chan et al., 2012), suggesting that other adhesins are crucial for adhesion and dissemination in this strain. An alternative explanation is that the recombinant protein used for *in vitro* studies may have different binding activities than the native borrelial protein.

Another group examined decorin binding of various genospecies including *B. afzelii*, *B. garinii*, and *B. burgdorferi* sensu stricto (Salo et al., 2011). There is only 40–60% similarity among the decorin binding proteins of these genospecies. Interestingly, *B. afzelii*, despite its preference for skin and its association with the Lyme disease skin manifestation known as acrodermatitis chronica atrophicans, doesn't bind decorin. The authors evaluated the binding abilities of both DbpA and DbpB independently. DbpA of *B. garinii*, as well as DbpB of *B. garinii* and *B. burgdorferi*, promoted strong binding to decorin. In contrast, DbpA of *B. burgdorferi* and DbpA and DbpB of *B. afzelii* exhibited lower binding to decorin. Despite differences in their binding to decorin *in vitro*, both DbpA and DbpB of *B. garinii*, as well as *B. burgdorferi*, promoted adhesion of whole spirochetes to decorin or decorin-expressing human foreskin fibroblasts (Salo et al., 2011). The authors suggest that the Dbps of *B. afzelii* have different biological functions and ligands than the Dbps of *B. garinii* or *B. burgdorferi*.

In a previously mentioned study with BBK32, luciferase-expressing *B. burgdorferi* lacking *dbpAB* were also injected into mice, and mutants were only visible at 1 h post infection (Hyde et al., 2011), suggesting efficient clearance of the spirochetes. These data again demonstrate a role for decorin-binding proteins in the initial colonization by *B. burgdorferi*.

Another group demonstrated that DbpA/B mutants have decreased infectivity and an early dissemination defect in immunocompetent but not immunodeficient mice. Development of arthritis and carditis decreased only in early stages of infection with DbpA/B mutants in either strain of mice. Up to 14 days post infection, the bacterial load in the lymph nodes of mice infected with DbpA/B mutants was decreased compared to lymph nodes of mice infected with wild type bacteria. The authors postulate that the immune response restricts early dissemination through the lymphatic system, which suggests a role for nonantibody mediated clearance, perhaps by iNKT cells. A particular adhesin may not be essential for infection, but can still influence pathogenicity by altering the course of infection through changing the ability to disseminate, colonize, or persist (Imai et al., 2013).

Finally, anti-DbpA antibodies were shown to significantly reduce the ability of whole *B. burgdorferi* to bind to H4 (neuroglial) and HUVEC cells. Indeed, *dbpA* expression was increased when *B. burgdorferi* was incubated with either cell type (Schmit et al., 2011). Overall, the data amassed in the last 3 years demonstrate that decorin binding proteins of *B. burgdorferi* are important in both the initial establishment of infection and persistence of the Lyme spirochete.

#### BbhtrA

The proteoglycan aggrecan is found in the ECM and cartilage, particularly articular cartilage of joints. Aggrecan is comprised of three globular domains, with 2 sites for GAG attachment. The N terminus of aggrecan binds hyaluronan, which is important for linking of aggrecan molecules to form aggregates (Watanabe et al., 1998). Aggrecan thus adds to the resiliency of functional cartilage. *B. burgdorferi* binds aggrecan, and both BbhtrA and Bgp were recently identified as aggrecan-binding proteins (Russell and Johnson, 2013). BbhtrA is significant as the first identified outer surface protease of *B. burgdorferi*. BbhtrA is expressed during human disease, is immunogenic, and is conserved among *B. burgdorferi* strains. BbhtrA both binds and cleaves aggrecan at a site known to eradicate its function, suggesting that this protease may contribute to the damage seen in Lyme arthritis (Russell and Johnson, 2013). Proteolytic activity for BbhtrA against *B. burgdorferi* proteins BB0323, BmpD, and CheX was also demonstrated, in addition to cleavage of host ECM proteins (Coleman et al., 2013; Kariu et al., 2013; Russell et al., 2013).

### Collagen-binding proteins

Collagens are triple helical proteins and the most abundant ECM proteins. Collagens are important structural components of many tissues, allowing tissues to withstand stretching (Lodish and Darnell, 2000). There are numerous collagens and related proteins. Collagens I, II, and III are the major fibrous collagens, while collagen IV is a major constituent of basement membranes (Labat-Robert et al., 1990). *B. burgdorferi* has long been known to bind collagen *in vitro*, but until recently no collagen binding adhesin had been identified (Zambrano et al., 2004). Hallstrom and colleagues recently demonstrated that CspA (CRASP-1), a factor H binding protein, also binds to collagen I, III, and IV (Hallstrom et al., 2010). More studies are necessary to elucidate the multiple roles of CspA in *B. burgdorferi* pathogenesis. Given the spirochete's redundant interactions with other ECM proteins, it is likely that other collagen binding adhesins remain to be discovered.

### Laminin-binding proteins

Laminin is a glycoprotein component of the ECM consisting of α, β, and γ subunits that self-assemble into a heterotrimer. Laminin serves a scaffolding function and interacts with integrins as well as other matrix components (Colognato and Yurchenco, 2000). *B. burgdorferi* binds laminin through BmpA and ErpX (Brissette et al., 2009c; Verma et al., 2009). Recently, the promiscuous CspA and CspZ proteins were also shown to bind laminin *in vitro* (Hallstrom et al., 2010).

### Integrin-binding proteins

Integrins are transmembrane bidirectional signaling molecules involved in cell-cell signaling and cell-matrix interactions. Intergins serve as ECM ligands and partner with many multiadhesive proteins including fibronectin, laminin, and collagen (Takada et al., 2007). These heterodimeric proteins consist of both α and β subunits. *B. burgdorferi* binds αIIβ3 (Coburn et al., 1993), αvβ3, α5β1 (Coburn et al., 1998), as well as α3β1 integrins (Behera et al., 2006).

*B. burgdorferi* binds α1β3 integrins through the P66 outer membrane protein and porin (Lafrance et al., 2011). Because of the ability of integrins to signal “outside-in,” the authors examined the effects of P66 on gene expression by human cells in culture. *B. burgdorferi* strains with and without P66 were incubated with EA-hy926 (endothelial) cells or human embryonic kidney cells (HEK 293) and subjected to microarray analysis. For both cells, but especially the endothelial cells, there were dramatic changes in the expression of ECM interacting proteins, components of the immune response, and genes encoding proteins involved in actin dynamics. The authors postulate that P66 may decrease the endothelial cell response, which in turn affects the ability of the endothelium to respond to the bacterial threat (Lafrance et al., 2011).

ΔP66 strains were not infectious in wild type, TLR2-, or MyD88- mice, suggesting that even in the absence of innate immune signaling, P66 is required to establish infection (Ristow et al., 2012). Interestingly, restoration of P66 on a shuttle vector did not restore infectious ability, but complementation on the chromosome did, supporting the hypothesis that transient adhesion is important (i.e., the overexpressing complemented strain was not infectious). ΔP66 mutants were cleared from site of inoculation, but survived in rat dialysis chambers. These data demonstrated that the inability to infect mice was not due to a nutritional defect, an important point as P66 is a porin as well as an adhesin. P66 was not required for survival in ticks, as ΔP66 mutants survived in the tick through the molt from larvae to nymph. Increased mast cells were present at the site of inoculation of ΔP66 strains, and the mutants did not show increased susceptibility to serum. The authors suggest that P66 binding to integrins is necessary to move from the site of inoculation to distant tissues (Ristow et al., 2012). The exact connection between the lack of P66 and bacterial clearance remains to be established.

Integrins were also shown to be involved in invasion of human endothelial cells and mouse fibroblasts by *B. burgdorferi*. This process required β1 integrins but not α5β1 or the fibronectin binding protein BBK32. Internalization did require actin reorganization, as well as Src kinase activity (Wu et al., 2011).

Wood et al. recently described a novel integrin binding factor (Wood et al., 2013). The protein encoded by *bb0172* was shown to be associated with the outer membrane by proteinase K digestion and Triton X-114 extraction. This protein bound α3β1 integrin, and has a von Willebrand factor (vWF) domain, which is oriented to the extracellular environment. vWF domains are important in both cell adhesion and protein-protein interactions (Schneppenheim and Budde, 2011). Changes in temperature affected *bb0172* expression, which was increased during the change from unfed to fed tick conditions *in vitro*. BB0172 was shown to be a metal ion-dependent integrin binding protein, and this paper marks the first report of a metal ion-dependent adhesion site (MIDAS) motif in *Borrelia* species. Of particular note from this study is the use of an *in vitro* system to determine the orientation of transmembrane helices in microsomal membranes; this may be a helpful tool for studying other helical membrane proteins of *B. burgdorferi* and other spirochetes (Wood et al., 2013).

### Plasminogen-binding proteins

Plasmin is a serine protease synthesized as the inactive zymogen plasminogen. Plasminogen is activated by physiological activators uPA (urokinase plasminogen activator) or tPA (tissue plasminogen activator) to the active serine protease plasmin. Plasmin's main role *in vivo* is to degrade fibrin-containing thrombi, but it can also degrade ECM components (Castellino and Ploplis, 2005). Plasminogen is abundant in serum and extracellular fluids, and is a favorite target of many pathogenic bacteria. Binding plasminogen to the surface of a bacterium can provide a potent protease that can assist in dissemination and invasion of the pathogen (Bhattacharya et al., 2012; Sanderson-Smith et al., 2012). Several plasminogen-binding proteins have been identified in *B. burgdorferi*, including OspA, OspC, ErpA/C/P, CspA, CspZ, BBA70, enolase, and a 70 kDa protein (Fuchs et al., 1994; Hu et al., 1997; Lagal et al., 2006; Brissette et al., 2009b; Hallstrom et al., 2010; Koenigs et al., 2013; Hammerschmidt et al., 2014). Plasminogen deficient mice have decreased spirochetemia upon *B. burgdorferi* infection (Coleman et al., 1997). Plasmin on the surface of *B. burgdorferi* facilitates movement through endothelial cell monolayers and degrades components of the ECM (Coleman et al., 1995, 1999). Clearly, acquisition of plasminogen is vital for efficient dissemination of *B. burgdorferi*.

#### Enolase

Enolase is a highly conserved glycolytic enzyme that should be cytosolic, as it lacks classical cell sorting sequences and cell anchoring moieties. Yet, enolase is a prime example of a “moonlighting” protein (Jeffery, 1999) that is found on the surface of a variety of eukaryotic cells where it can function as a plasminogen receptor (Pancholi, 2001). A number of bacteria express enolases, which likewise can be found on the surface of bacterial cells and function as plasminogen receptors (Pancholi and Fischetti, 1998; Lahteenmaki et al., 2001; Pancholi and Chhatwal, 2003).

Three research groups published that *B. burgdorferi* enolase, BB0337, is a plasminogen-binding protein (Floden et al., 2011; Nogueira et al., 2012; Toledo et al., 2012). Floden et al. demonstrated that this binding was dependent on lysine residues but not influenced by ionic charge. Enolase was localized to the outer surface as shown by proteinase K digestion and immunogold electron microscopy. Plasminogen bound to *B. burgdorferi* enolase was converted to active plasmin by the plasminogen activator uPA (Floden et al., 2011). Noguiera et al. also showed that BB0337 was associated with the outer membrane and surface exposed. BB0337 retained its enzymatic activity intrinsic to the glycolytic pathway, and interacted with plasminogen via lysine residues. The *B. burgdorferi* enolase showed variable temporal and spatial expression in ticks, with expression relatively high in fed and unfed nymphs. In a mouse model of infection, enolase demonstrated its highest expression in joints and heart at 28 days post-infection, while expression in skin disappeared by day 28. Immunization with BB0337 did not evoke protective immunity in mice, but did prevent acquisition by ticks (Nogueira et al., 2012). In contrast, a third group found BB0337 in outer membrane vesicles, but did not detect enolase on the outer surface of *B. burgdorferi* by proteinase K digestion or by electron microscopy (Toledo et al., 2012). In addition, Toledo et al. demonstrated that enolase was recognized by host antibodies from tick-infected mice, rabbits, and human Lyme disease patients (Toledo et al., 2012).

#### OspC

In a recent study, Önder et al. make the case for OspC as the principal plasminogen-binding protein of *B. burgdorferi* (Lagal et al., 2006; Onder et al., 2012). The authors demonstrated that OspC bound plasminogen *in vitro*, and also showed co-localization of plasminogen and OspC on the surface of intact bacteria. Finally, OspC-deficient bacteria did not appreciably bind plasminogen, and plasminogen binding was effectively blocked by anti-OspC antibodies (Onder et al., 2012). The authors note that these results do not preclude a role for the other identified plasminogen-binding proteins under different conditions.

#### CspA (CRASP-1)

CspA binds plasminogen *in vitro*, and this interaction was inhibited by 6-ACA, a lysine analog. In the presence of uPA, plasminogen bound to CspA was converted to active plasmin (Hallstrom et al., 2010).

#### BBA70

Recently, Koenigs et al. described another plasminogen binding protein of *B. burgdorferi*, BBA70 (Koenigs et al., 2013). As with other plasminogen binding proteins, the interaction between BBA70 and plasminogen was dose-dependent and affected by ionic strength. This interaction was mediated by lysine residues in the C-terminus of BBA70. The authors demonstrated that BBA70 is located on the borrelial outer surface. Plasminogen bound to BBA70 was converted to active plasmin by urokinase-type plasminogen activator, and was able to degrade fibrinogen. Interestingly, BBA70-bound plasmin was able to degrade the central complement proteins C3b and C5 (Koenigs et al., 2013).

### Complement regulator acquiring surface proteins

Factor H is a 155 kDa protein present in mammalian serum that is important for homeostasis of the complement system. Factor H binds C3b, and accelerates the decay of the alternative pathway C3 convertase C3bBb, as well as serving as a cofactor for Factor I-mediated proteolytic inactivation of C3b (Rodriguez de Cordoba et al., 2004). Factor H consists of 20 short consensus repeats (SCRs); SCRs 19–20 interact with GAGs, such as heparin, on the surface of host cells.

There are additional Factor H-like proteins; Factor H-like protein-1 (FHL-1) is encoded by the same gene, but is a product of alternative splicing of the Factor H pre-mRNA. Factor H related proteins (FHRs) are encoded by separate genes but have sequence similarities with SCRs 18–20 of Factor H. FHR-1 blocks the action of the C5 convertase, as well as the membrane attack complex (MAC) formation, while FHR-5 has cofactor activity for Factor I-mediated inactivation of C3b (McRae et al., 2005). The function of FHR2 is unknown.

*B. burgdorferi* has several proteins which bind Factor H and its related proteins, initially identified as CRASPs (Complement Regulator-Acquiring Surface Proteins), including CRASP-1 (CspA), CRASP-2 (CspZ), and CRASP-3 (ErpP), CRASP-4 (ErpC), and CRASP-5 (ErpA) (reviewed in Kraiczy and Stevenson, 2013). Many of these proteins have multiple designations in the literature; to avoid confusion, the designations CspA, CspZ, ErpP, ErpC, and ErpA will be used here.

A number of exciting biochemical and biophysical studies recently have provided important insights into the interaction of borrelial Factor H-binding proteins with their ligands. One study examined the structural basis for complement evasion. The authors solved the solution structure of ErpP interacting with Factor H by NMR and X-ray crystallography. Factor H SCR 19–20 interacts with ErpP, reminiscent of how Factor H binds to GAGs (Bhattacharjee et al., 2013). Another study resolved the atomic resolution structure of ErpC. This protein consists of 10 antiparallel β strands capped by two α helices. The outer surface is charged, while the inner surface is hydrophobic. ErpC bound Factor H through residues within the loops between the β strands (Caesar et al., 2013a). In contrast, CspA, which forms homodimers, was shown to consist of 7 α helices joined by short loops. The flexibility between the subunits may allow for increased access of Factor H to the binding site. The C terminus of CspA is required for assembly of a stable dimer (Caesar et al., 2013b). Finally, Meri et al. showed that ErpA of *B. burgdorferi*, as well as Factor H binding proteins from the relapsing fever spirochete *B. hermsii* and other pathogens, binds Factor H through a common site in SCR 20. The authors showed that binding of Factor H by these pathogens was inhibited by heparin binding, and that a tripartite complex between the microbial protein, Factor H and C3b is formed, facilitating complement evasion (Meri et al., 2013).

The contribution of the Factor H-binding proteins to complement evasion is being heavily investigated not just for *B. burgdorferi*, but also *B. afzelii* and *B. spielmannii*. Kenedy and Akins overexpressed ErpP and ErpA in a *cspA*-deficient strain. The overexpression of these proteins enhanced serum resistance and the amount of Factor H bound to the bacterial surface. Deposition of complement components C3 and C5b-9 (the MAC complex) were reduced on the surface when ErpP or ErpA were overexpressed (Kenedy and Akins, 2011).

ErpC immobilized on magnetic beads captured Factor H, as well as FHR-1, -2, and -5 from human serum. However, *B. garinii*, which is sensitive to complement killing, expressing ErpC were still killed by complement (Hammerschmidt et al., 2012), suggesting ErpC alone is insufficient to protect the Lyme bacterium from complement.

*Borrelia spielmanii* binds both Factor H and plasminogen, and is serum resistant. *B. spielmanii* has a 15 kDa CRASP protein (akin to the 17–20 kDa ErpA, C, and P proteins of *B. burgdorferi*) that was shown to bind Factor H, FHR-1 but not FHL-1. Factor H bound to BsCRASP maintained its cofactor activity for Factor I-mediated C3b inactivation. Mutating H79 to alanine abrogated Factor H binding but not plasminogen binding, demonstrating independent binding sites on BsCRASP for plasminogen and Factor H. *B. spielmanii* also expresses a CspA-like protein (Seling et al., 2010).

Finally, *B. burgdorferi* ErpP and ErpA bind FHR-2 and FHR-5. In contrast, *B. garinii* CRASPs bind FHR-1, -2, and -5 but not Factor H. ErpA, ErpC, and ErpP bind Factor H and FHR-1, but not FHL-1. The binding properties of ErpA, ErpP, and ErpC are different for the recombinant proteins as compared to the native proteins. *B. burgdorferi* lacking CspA and CspZ, which bind both Factor H and FHL-1, were killed by complement. The balance of the data suggest that the Lyme *Borrelia* require acquisition of Factor H, but not FHRs, to evade complement-mediated killing (Siegel et al., 2010). A recent review by Kraiczy and Stevenson neatly summarizes the acquisition of Factor H, FHL-1, and FHRs by the various *B. burgdorferi* proteins (Kraiczy and Stevenson, 2013).

## Concluding remarks

Although much progress has been made in the field, further work is needed to elucidate the roles of the many identified borrelial ECM-binding proteins and other adhesins (Table 1). Obstructing progress are several characteristics inherent to these types of proteins. For instance, redundancy for specific host substrates can prevent phenotypic characterization of adhesin mutants, as the lack of one protein may be compensated for by the presence of several others. Another obstacle for research is the ability of multiple borrelial proteins to each interact with several substrates. Defining a role for a given protein during an infection may be difficult when that protein may fulfill different functions during the course of an infection. Compounding these issues are the different potential roles for every protein in the infection process of the bacteria. To be infectious, *B. burgdorferi* must colonize, disseminate, and persist in the host, all of which are facilitated by proteins discussed herein. To this end, these studies highlight the need to examine adhesins *in vitro*, *in vivo*, and with both loss and gain of function mutants. Determining which stage or stages of infection each protein participates in will help us to further understand the biology of the bacteria and potentially allow for novel treatment strategies for Lyme disease. For example, anti-adhesive therapies that target the initial interactions between bacteria and their host are garnering considerable interest in this era of antibiotic resistance (Krachler and Orth, 2013). Once the precise roles of these sticky *B. burgdorferi* proteins are well understood, these adhesins themselves may prove to be responsive targets for Lyme disease therapies.

### Conflict of interest statement

The authors declare that the research was conducted in the absence of any commercial or financial relationships that could be construed as a potential conflict of interest.

## Methods

This review covers new research published on *B. burgdorferi* adhesins from January 2010–October 2013. Search terms on PubMed: Borrelia + adhesin, Borrelia + fibronectin, Borrelia + laminin, Borrelia + decorin, Borrelia + integrin, Borrelia + collagen, Borrelia + proteoglycan, Borrelia + GAG, Borrelia + Factor H, Borrelia + plasminogen.
